# Editorial: The problem of childhood hypoglycemia, volume II

**DOI:** 10.3389/fendo.2024.1412976

**Published:** 2024-04-29

**Authors:** Indraneel Banerjee, Klaus Mohnike

**Affiliations:** ^1^Department of Paediatric Endocrinology, Royal Manchester Children’s Hospital, Manchester University Hospitals National Health Service (NHS) Foundation Trust, Manchester, United Kingdom; ^2^Faculty of Medicine, Otto von Guericke University Magdeburg, Magdeburg, Germany

**Keywords:** hypoglycemia, congenital hyperinsulinism (CHI), adrenal insufficiency, continuous glucose monitoring (CGM), Katp (Kir6.2) channel, small for gestation age (SGA), magnetic resonance imaging (MRI), neonate

Hypoglycemia is common in childhood with long-term neurodisability from irreversible neuronal injury; yet the problem remains obdurately unresolved. The Research Topic series “*The Problem of Childhood Hypoglycemia*” provides an overview of different aspects from translational medicine to patient experience, reflecting a diversity of views from endocrinology, metabolism, genetics, neonatology, radiology and psychology integrated into a multidisciplinary assessment of hypoglycemia.

Despite significant research inquiry, a clear definition of neonatal hypoglycemia has not emerged (Roeper et al.). Mathematical definitions of hypoglycemia, supported by validating studies, have been advocated but they disregard individual variability and the differential effects of the duration and excursion of hypoglycemia. Symptom-based definitions are also ineffective as hypoglycemia is often asymptomatic. The traditional emphasis on glucose and ketones as hypoglycemia metabolites has impeded a search for biomarkers correlating with brain injury, an outcome resonating with a brain magnetic resonance imaging (MRI) study documenting missed neonatal hypoglycemia (Worth et al.). As with data of a non-converging nature, larger studies have been proposed to generate longitudinal neurodevelopmental outcomes to magnify small scale effects (Roeper et al.). However, such studies are cumbersome and predicated on current insufficient understanding, suggesting the need for unbiased hypothesis-free study designs.

While the debate on neonatal hypoglycemia continues unabated, it is important to optimize therapeutic strategies that mitigate against the ill-effects of neuroglycopenic injury, as in the rare disease of congenital hyperinsulinism (CHI) (**Figure 1**). A standardized practice guideline by specialist centers and utilizing a network model serves as a template to harmonize investigations and treatment plans for CHI patients (Shaikh et al.). This guideline also offers multidisciplinary practical advice on day-to-day parent perspectives such as breast feeding, weaning and follow-up to give a more holistic view of clinical management.

**Figure 1:**
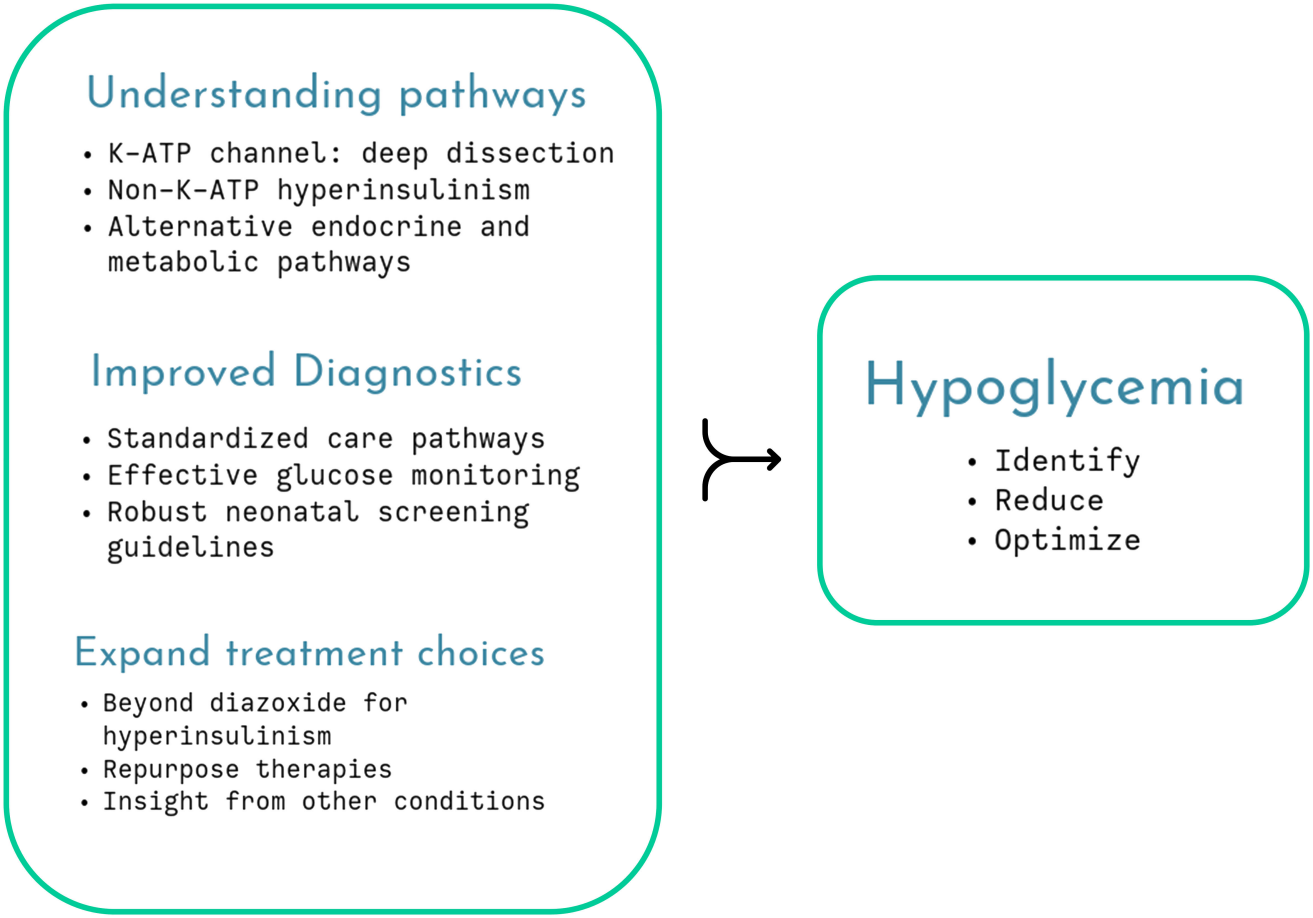
Several aspects of childhood hypoglycemia remain unresolved but cumulative research will unravel understanding, enabling improved therapy and monitoring, optimizing glycemic status.

A return of emphasis on the identification on neonatal hypoglycemia (Worth et al.) is presented in a ten-year review of brain MRI. The study identified characteristic radiological features of hypoglycemia in children without a history of neonatal hypoglycemia. Although the study was not designed to establish the true prevalence of missed hypoglycemia, the finding of any missed hypoglycemia escaping from standard neonatal screening procedures is concerning.

An important paradigm that inevitably influences the management of hypoglycemia is the monitoring of glucose levels. In most cases, local investigation algorithms mandate infrequent blood glucose monitoring that fail to account for continuously variable glucose profiles captured by continuous glucose monitoring (CGM) devices. CGM is increasingly recognized in the management of childhood hypoglycemia, albeit with caveats. A comprehensive review of the benefits and challenges in CGM predicts a role for the monitoring of both neonatal hypoglycemia and hyperglycemia (Worth et al.). However, the evidence for wider adoption in childhood hypoglycemia is not strong and calls for rigorous research not only in CHI but also in other disorders such as glycogen storage disorder (GSD) and adrenal insufficiency (Lee et al.).

Considerable attention has been devoted to the understanding of genetic forms of CHI, but less to non-genetic forms of hyperinsulinism, for instance in small for gestational age (SGA) newborn infants. A relatively large observational cohort investigated if diazoxide has greater clinical benefit than a wait/watch approach for hyperinsulinism in SGA (Chandran et al.). Surprisingly, the use of diazoxide did not reduce length of stay in the hospital, although both treatment strategies involved unusually prolonged hospitalization. The authors conclude that watch/wait is as effective as diazoxide, accepting the limitations of variability in a real-world study without longitudinal follow-up to determine neurodevelopmental equivalence.

Another relatively unrecognized cause of non-genetic hyperinsulinism is congenital porto-systemic shunts (CPSS) (van Albada et al.). In a review, illustrated by a case report, the authors present well-argued theoretical constructs describing hepatic bypass as a cause for a surge in insulin and reduced degradation causing hypoglycemia. Treatment for symptomatic CPSS include endovascular or surgical approaches, but slow-release carbohydrates may also prove effective. As with the cautionary narrative of diazoxide in SGA hyperinsulinism (Chandran et al.), diazoxide may increase glucose levels, precipitating post-prandial hypoglycemia in CPSS.

Not surprisingly, the topic dwells on mechanistic insights, both involving (ElSheikh and Shyng) and excluding (Chen and Sang) the pancreatic β-cell K-ATP channel to unravel the pathophysiology of CHI. The K-ATP channel is a central mechanism explaining glucose-insulin coupling ensuring appropriate insulin secretion based on need. Pathogenic variants in the genes coding for the subunits of the channel, *ABCC8*/*KCNJ11*, variably affect channel gating, protein folding, assembly and trafficking, providing a deeper understanding of phenotypic diversity (ElSheikh and Shyng). The authors stress the importance of molecular characterization, gaining insight from novel missense and indel variants as well as non-coding regions to design future therapeutic targets. They suggest laboratory simulation to test dominant effects and pathogenicity and use the latest electron microscopy techniques to describe channel structure in vivid detail but accept that phenotypic variation cannot always be simulated by experiments. Nonetheless, careful dissection of the K-ATP structure and function may eventually identify treatments such as pharmacological chaperones (**Figure 1**).

In CHI, diazoxide remains first line treatment, although unresponsiveness is common. In a small group of patients with diazoxide-unresponsive CHI, K-ATP pathogenic variants were found in 69%, but no genetic etiology was ascertained in the remainder (Lee et al.). Preponderance of a specific Taiwanese variant, probably due to a founder effect, was noted and characterized by functional studies demonstrating loss of channel function.

A rare but interesting etiology of CHI has been described in a review on variants in phosphomannomutase 2 (PMM2) (Chen and Sang). *PMM2* variants contribute to abnormal protein glycosylation, including the sulfonylurea receptor (SUR), thereby modulating insulin secretion. Although PMM2 hyperinsulinism has been reported in only 26 patients, mechanistic insight may improve our overall understanding, including the role of *HNF4A*, a known CHI-related gene.

Hypoglycemia in adrenal insufficiency has not received much research attention, even though it is a well-recognized component of life-threatening adrenal crisis in both primary and secondary forms of the condition. A timely review (Lee et al.) summarizes mechanisms of hypoglycemia through loss of counter-regulation, compounded by reduced epinephrine stores and the presence of other hormone deficiencies. The review notes that adrenal hypoglycemia is probably underreported, given the relative high incidence of adrenal crisis in young children. It advocates the need for improved medications and considers glucose monitoring with CGM as a future adjunct to clinical management (**Figure 1**).

Empagliflozin used in adults with type 2 diabetes has been repurposed for application in the hypoglycemia disorder of GSD (Maiorana et al.). Empagliflozin inhibits renal glucose transporters, preventing glucose reabsorption and reducing glucose levels. In GSD type 1b, accumulation of 1,5 anhydroglucitol interferes with metabolic pathways within neutrophils, causing neutropenia. Here, empagliflozin induced glycosuria facilitates greater excretion of 1,5 anhydroglucitol to preserve neutrophil function. The authors describe clinical benefit in GSD1b, although without direct improvement of hypoglycemia; instead, they seek insight from protective pleiotropic effects on the heart, kidney, liver and neurons in type 2 diabetes, thereby utilizing collateral experiences in the repurposing of medications.

## Author contributions

IB: Conceptualization, Writing – original draft, Writing – review & editing. KM: Conceptualization, Writing – review & editing.

